# Pancreatic Cystic Lesions: Pathogenesis and Malignant Potential

**DOI:** 10.3390/diseases6020050

**Published:** 2018-06-13

**Authors:** Antoinette J. Pusateri, Somashekar G. Krishna

**Affiliations:** 1 Department of Internal Medicine, The Ohio State University Wexner Medical Center, Columbus, OH 43210, USA; Antoinette.Pusateri@osumc.edu; 2 Division of Gastroenterology, Hepatology, and Nutrition, The Ohio State University Wexner Medical Center, Columbus, OH 43210, USA

**Keywords:** pancreatic cystic lesions, mucinous lesions, intraductal pancreatic mucinous neoplasm, mucinous cystic neoplasm, solid pseudo-papillary tumors, cystic neuroendocrine tumors, pancreatic ductal adenocarcinoma

## Abstract

Pancreatic cancer remains one of the most lethal cancers despite extensive research. Further understanding of precursor lesions may enhance the ability to treat and prevent pancreatic cancer. Pancreatic cystic lesions (PCLs) with malignant potential include: mucinous PCLs (intraductal papillary mucinous neoplasms and mucinous cystic neoplasm), solid pseudopapillary tumors and cystic neuroendocrine tumors. This review summarizes the latest literature describing what is known about the pathogenesis and malignant potential of these PCLs, including unique epidemiological, radiological, histological, genetic and molecular characteristics.

## 1. Introduction

Pancreatic cancer is one of the most lethal cancers. In the United States, pancreatic cancer is projected to become the second leading cause of cancer-related deaths by 2030, and currently there are no methods in practice for early diagnosis [1]. The lethality of pancreatic cancer is the worst of all cancers analyzed, in that across all races, the five-year survival for all stages is 8% [2]. This is thought to be due in part to the fact that 52% of subjects present with metastatic disease, a stage which itself has a 3% five-year survival rate [2]. While there is the potential to make an earlier diagnosis of a similarly late-presenting cancer such as lung cancer with low-dose computed tomography (57% of cases presenting as distant disease), there is no such test for early detection of pancreatic cancer at this time [2].

One strategy for early detection of pancreatic cancer could be prevention through the identification of known precursor lesions. Pancreatic cystic lesions (PCLs) with malignant potential include: mucinous PCLs [intraductal papillary mucinous neoplasms (IPMNs) and mucinous cystic neoplasm (MCNs)], solid pseudopapillary tumors (SPTs), and cystic neuroendocrine tumors (cystic-NETs). The benign lesions include: serous cystadenoma (SCA), pseudocysts, and squamous epithelium lined PCLs (lymphoepithelial cysts, epidermoid cysts). Incidental PCLs are diagnosed in 13–45% of patients undergoing MRI and 2% of CT scans [3,4]. Given that PCLs exist on a spectrum of benign to malignant potential, they have been the subject of much review and debate regarding clinical management [5,6,7,8,9].

This review will focus on the unique epidemiological, radiological, histological, molecular and genetic aspects of PCLs with malignant potential in order to understand their risk of progression, and, therefore, ability to potentially prevent pancreatic cancer. The use of diagnostic modalities such as endoscopic ultrasound (EUS) being the standard of care for management of PCLs, is beyond the scope of this review.

## 2. Intraductal Papillary Mucinous Neoplasms (IPMNs)

IPMNs represent 21–33% of PCLs and have been described to be equally prevalent in men and women, if not slightly more in men, occur in the 6th to 7th decade of life, preferentially in the pancreatic head as opposed to the body or tail [10,11,12,13]. Macroscopic location divides IPMNs into three duct types: main duct (MD), branch duct (BD) and a combination of these called mixed duct. Of the four PCL types discussed in this review, IPMNs bear the greatest malignant potential, with up to 27.6–68% progression to invasive carcinoma [12,14,15]. Notably, invasive IPMNs still have a comparatively better prognosis than pancreatic ductal adenocarcinoma (PDAC), with 5-year survival rates ranging between 40 and 60% in various studies [12,16,17]. Malignant progression of IPMNs can occur over the course of many years, however, and specific radiologic, microscopic, molecular and genetic features can help predict this [18]. 

IPMN location, type, and dysplastic changes are a function of histological type characterized as either intestinal, oncocytic, pancreaticobiliary or gastric. Furthermore, dysplasia within these types can be further characterized by invasion subtype, including colloid (also referred to as mucinous), tubular (also referred to as ductal), or oncocytic carcinomas [17]. This is relevant because each combination of these histologic types and invasion subtypes has prognostic implications [18,19] (Table 1, Supplementary Material).

Of the MD-IPMN, intestinal type IPMN is the most common and occurs in the pancreatic head. It is villous in morphology and typically has a mucin protein expression pattern of MUC1-, MUC2+, MUC5AC+. Furthermore, the majority of intestinal type IPMNs express CDX2, a tumor suppressor implicated in intestinal differentiation. Its invasive subtype is usually colloid and has been described to occur at a rate of 41.6% in one study [17,20,21,22].

Pancreaticobiliary type IPMN is rarer than the intestinal type but is also found in MD-IPMNs and occurs in the pancreatic head. In contrast to the intestinal type, it is typically MUC1+, MUC2-, and MUC5AC+ and does not express CDX2 [18]. This is notable because MUC1 has been found to have an inhibitory role in cell–cell interactions, immunoresistance, and to aid progression of carcinoma cells, thus clinically serving as a marker of more aggressive phenotype in PDAC [23]. MUC2 has been shown to serve as a tumor suppressor in mice and creates a mucin protein that is a protective barrier in the intestinal epithelium. CDX2 has been shown to support MUC2 in its tumor suppressor activity and is not usually expressed in PDAC [20,24,25,26,27]. In contrast to the intestinal type, the invasive subtype in the pancreaticobiliary type is usually tubular and was described to have an invasion rate of 63.2% [17,20,21,22].

The oncocytic type IPMN also occurs in MD-IPMNs, mostly in the pancreatic head, expresses MUC1+, MUC2-, and MUC5AC+, and exhibits high-grade dysplasia; however, it is exquisitely rare, produces little mucin relative to the other types, and similar to other tumors with increased intra-cytoplasmic mitochondria, appears to have a different biological pathogenesis. Although one study describes an invasion rate of 45.8%, it typically has an indolent course with a favorable prognosis [18,22,27,28,29,30,31,32].

Considering MD and BD IPMN together, the most common type of IPMN overall and the most common type of BD-IPMN is the gastric type. The gastric type is found in the uncinate process and periphery of the pancreas, expresses MUC1-, MUC2-, MUC5AC+, and MUC6+, and can possess severe cellular atypia and invasion, with invasion rates described at 9.4% [18,22]. One study demonstrated that when the gastric type becomes invasive, it develops into tubular carcinomas similar to the pancreaticobiliary type [15]. However, other studies have shown that the gastric type usually does not exhibit malignant potential [15,19,21]. Gastric type IPMNs have also been shown to occur in association with pancreatic intraepithelial neoplasia (PanIN) 1 (another MUC5AC+ lesion). This is connected on the molecular level by the finding that pancreatic ductal epithelium responds to sonic hedgehog signaling to form gastric mucinous metaplasia and PanIN-like formation [33]. Thus, the gastric type IPMN could bear a higher predisposition to PDAC [18].

These histological, molecular, and genetic findings are consistent with the prognostic findings of several studies. Several retrospective analyses have demonstrated that IPMN type is strongly associated with patient survival, even above macroscopic type or histologic staging (Table 1) [15,22]. With that being said, Distler et al. found that size rather than tubular invasive subtype was predictive of malignancy [15]. Nonetheless, in their study, pancreaticobiliary type was shown to be an invasive tubular subtype in 90.2% of cases with an overall 5-year survival rate of 35.6% [15] to 52.0% [22]; for comparison, 5-year survival for PDAC in the latter study was 11.1%. The gastric type was invasive tubular subtype in 30.8% of cases [15]; the overall 5-year survival was 70% [15] to 93.7% [22]. The oncocytic type was invasive oncocytic (not tubular or colloid) subtype in 75% of cases, had an overall 5-year survival of 75% [15] to 83.9% [22], however this was in a sample of 4 subjects. One study even showed that it had a 100% 5-year survival rate [32], although as one meta-analysis pointed out, given the rarity of the oncocytic type and small number of long-term follow up, it remains unclear if the prognosis of the oncocytic type is truly superior to other types [31]. The intestinal type was invasive colloid subtype in 55.6% of cases with an overall 5-year survival of 86.6% [15] to 88.6% [22].

Overall, low-grade dysplasia has a statistically higher disease-specific survival rate than high-grade dysplasia, and invasive IPMN has the worst disease-specific survival, with tubular being worse than colloid [13]. Tubular carcinoma has been shown to be statistically associated with lymph node metastasis, perineural invasion, vascular invasion as well as tumor size and positive margins versus colloid carcinoma. Colloid invasive carcinoma bears a better 5-year survival rate than its tubular counterparts, to the order of 60–70% and 40–50%, respectively [12,16,17,18,21]. Overall recurrence of resected IPMN has been shown to be low at 0–11% in one review and 14.1% in one primary study [22,34], although another primary study described recurrence rates of up to 41.7% [15]. While Table 1 illustrates different recurrence rates for the various histological types [15,22], invasive status is important to note as well: patients with resection of non-invasive, high-grade dysplasia IPMNs have been shown to have a lower risk of metastasis or recurrence than resection of invasive IPMNs, while resected non-invasive, high grade dysplasia IPMNs have been shown to have an increased risk of subsequent development to PDAC than those with resection of non-invasive low/intermediate-grade dysplasia [13]. It should be noted that the 2016 Baltimore Consensus Classification classifies dysplasia as either low or high, thus, intermediate dysplasia is now categorized under low dysplasia [35]. The presence of dysplasia at resection margins of non-invasive IPMN bears significant implications on recurrence: 2% with negative margins for IPMN recurred versus 17% with positive margins [34].

IPMNs may also be associated with PDACs. IPMNs and PDACs have been shown to follow an adenoma to carcinoma sequence, the former less aggressive and slow-growing and the latter more aggressive and rapidly growing [36]. Patients with resection for IPMN may also develop de novo PDAC [18], specifically derived from IPMNs in 16% of cases, concomitant with IPMNs in 4% of cases, and of unknown relation in 4% of cases in one retrospective review [37]. A more recent study, which demonstrated PDAC and co-occurring IPMN in 8% of patients found that co-occurring IPMN/PDAC lesions were associated with high-grade IPMN, gastric and pancreaticobiliary types of IPMN, and were likely related in 51% of cases [38]. With that being said, 18% were likely independent, and more than 80% of the IPMNs lacked mutations in the high-risk genes TP53 and SMAD4 that are usually detectable in PDAC [38].

IPMNs can be classified as single or multi-segmental, synchronous (having been removed at the same time) or metachronous (having grown into a distinct lesion after a resection with negative margins), or by genetic clonality [39]. A recent study found marked genetic heterogeneity in IPMN lesions, suggesting poly-clonality in these lesions [38]. Critical mutations include the *KRAS* oncogene codon 12 in 31–86% of IPMNs [18,36,40,41,42,43,44] compared to the *KRAS* oncogene codon 12, 13, and 16 in nearly 100% of PDACs [18,45]. *KRAS* mutations had the highest frequency at 100% in the pancreaticobiliary type [46] and tubular subtype at 89% [47]. 

Another critical oncogene is *GNAS*, as mutations in *GNAS* codon 201 are common and specific for IPMNs, found in 41–66% of specimens, but not typically present in classical PDAC [46,48]. GNAS mutations have been shown to be most prevalent in the intestinal type IPMN, ranging from 74–100% in several studies [38,39,46,47] and colloid subtype at 89% [47]. This is reinforced by a recent study, where IPMNs with colloid carcinomas revealed higher mutations in *GNAS* (82%) with lower *KRAS* (45%) alterations [38]. Furthermore, using *GNAS*, one study demonstrated that IPMNs might be characterized by monoclonal skip lesion progression. This is important not only for prognosticating on the IPMN itself, but also to distinguish invasive IPMN from classical PDAC since the latter rarely has *GNAS* mutations [39].

These mutations are important for distinguishing IPMNs from other pancreatic cystic lesions: while 91–99% of IPMNs have either *KRAS* and/or *GNAS* mutations without significant variation in degree of dysplasia [38,47], serous cystadenomas generally have neither mutation, and while not as sensitive, *GNAS* mutations can help distinguish IPMNs from mucinous cystic neoplasms, as will be discussed next [46].

## 3. Mucinous Cystic Neoplasm (MCN)

MCN make up 10% of pancreatic cystic lesions [49], and can be differentiated from others such as SPTs and cystic-NETS due to the presence of mucin-producing epithelium [50]. Distinction from mucin-producing IPMNs makes this more challenging but can be elucidated through epidemiological and macroscopic features such as lack of communication with the pancreatic ductal system, location in pancreatic body or tail, and predominately (90–95%) effecting women in their 4th decade of life [51].

Imaging with CT-Scan typically shows well-encapsulated, cystic masses often with septations dividing mucoid and hemorrhagic fluid contents as well as the presence of peripheral calcifications [52,53]. MCNs tend to be single lesions and are best known for the presence of ovarian-type stroma, versus IPMNs that are multifocal and have four main histological types as described earlier [54]. Ovarian-type stroma is defined as “densely packed spindle-shaped cells with round or elongated nuclei and sparse cytoplasm” [51]. MCNs disproportionately affect premenopausal women (mean age 47 years), and these lesions are estrogen- and progesterone-receptor-positive [50,55]. When compared to MCN of the ovary, MCN of the pancreas express similar rates of calponin, h-caldesmon, alpha-inhibin, estrogen receptor and progesterone receptor; some neoplastic epithelial cells of MCN of the pancreas even expressed human chorionic gonadotropin [56]. These data support the hypotheses that MCN of the pancreas form due to hormonal influences imparted by the embryological development of the distal pancreas in close proximity to the left ovary [50,57].

Compared to the aforementioned IPMNs invasion rate of 27.6–68%, MCNs are also invasive in up to about 17% of cases [54,55,58,59,60,61]. To directly compare to the IPMN types, one study demonstrated that while more than half of non-invasive MCN cases were MUC2+ or CDX2+, with 40% being MUC2+ and CDX2+, all invasive cases (*n* = 3) were MUC1+, MUC2- and only one case was CDX2+ [58]. As for invasive subtype, the MCN invasive carcinomas are predominantly of the ductal subtype, although some studies describe an undifferentiated/sarcomatoid carcinoma subtype as well; the colloid subtype described in IPMN is typically not associated with MCN [55,62].

Invasion has been shown to be associated with MCN size greater than 3 cm and presence of intracystic papillary nodules [55,60]. While overall 5-year survival for MCN has been reported to be 75–93% [55,59,61], it has been described as 100% for non-invasive and 26–57% for invasive carcinoma, although early invasion has been shown to have a better prognosis than advanced invasion [55,59]. A study of 16 minimally invasive MCNs defined as invasion confined to the ovarian stroma without capsular or pancreatic parenchymal invasion demonstrated that the majority of patients were cured by complete surgical resection [62]. Furthermore, another study showed that 4.5% of patients with resected MCN that had recurrence all had invasive carcinoma at initial resection [59]. 

In contrast to IPMNs, MCNs have been reported to not have *GNAS* mutations and fewer loss of heterozygosity (LOH) mutations [46,49]. In contrast to SPTs, MCNs typically do not have CTNNB1 mutations [63]. MCNs have been shown to possess *KRAS* mutations in 50–75% of specimens [49,63]. One study demonstrated that all MCNs with KRAS mutations were mutated at *KRAS* codon 12 [49]. Other mutations described include *RNF43*, which has been shown to be inactivated in IPMN as well and may be related to p53-mediated apoptosis. Few *TP53* mutations were shown in MCN, and may be associated with more aggressive behavior [49].

## 4. Solid Pseudopapillary Tumors (SPT)

Solid pseudopapillary tumors are rare, making up 1–2% of pancreatic neoplasms [64] and 5% of PCLs [49]. Although they have an 8–20% malignancy rate [5], they have an excellent prognosis following resection (95–100% 5-year survival rate) [61,65]. One review of the literature from 1933 to 2003 identified 718 patients reported to have SPT, almost all of whom were women, in their third decade of age, with lesions in the pancreatic tail or head. While only 69.22% of patients had information regarding metastases or invasion, 19.52% of them were positive for invasive disease. While follow up was only mentioned for 65.04% of patients and was limited to 5 years or less for 66.17% of those, 5-year survival was determined to be 95% [66]. One study documented a case of SPT that was initially thought to be a pancreatic pseudocyst, and no further work up or treatment was pursued until 13 years later when a palpable mass was discovered, which speaks to the slow progression of the tumor [67]. A review of the literature by this same group demonstrated that recurrence after radical resection of SPT occurs in 10–15% of cases, with the liver being the most common site of recurrence.

SPT can grow quite large (on average 9 cm). On CT scans they are encapsulated lesions with peripheral solid and central cystic components found to be hypo-attenuating in pancreatic and portal phases. MRI is helpful to analyze the hemorrhagic degeneration of the lesions. Smaller lesions of 3 cm in size tend to be without a capsule, more solid, and without hemorrhage, making them harder to differentiate from other solid pancreatic lesions and necessitating reliance on cellular and molecular findings [64].

The cellular origins of SPT are unclear. The neoplastic cells of SPT do not have a counterpart in the normal pancreas [49]. SPTs do not strongly stain for epithelial origin, the presence of vimentin is not consistent with pancreatic origin, and the presence of neuron specific enolase (NSE) but no other neuroendocrine markers suggests that SPT cannot be of pure neuroendocrine origin [68,69]. The presence of alpha-1 antitrypsin (A1AT) has been related to Mullerian mixed mesodermal tumors of the ovary. Given the predominance of SPT in women (similar to MCN) there may be a relationship between these lesions and embryologic development of the pancreas and left ovary [68]. On the other hand, the presence of differentiation proteins (A1AT, NSE) together with stem cell proteins (vimentin) could indicate origins from pluripotent pancreatic embryonic stem cells and neural crest embryonic neural precursor cells that undergo incomplete differentiation to form SPT [69,70,71].

There are counter-arguments to both theories. While some suggest SPT may arise from totipotent epithelial cells that differentiate into exocrine and/or endocrine elements [65], others suggest that the low proliferative activity and malignancy rate in SPT are more consistent with terminally differentiated cells than totipotent cells [68,70]. As for the embryologic theory, 0% of SPT in several studies were positive for estrogen receptor [66,68,70], and since these lesions still do occur in men, further investigation into the origins of SPT is still needed.

Nevertheless, SPTs are consistently described histologically as a combination of solid, pseudopapillary and/or hemorrhagic pseudocystic structures, all staining positive for PAS hyaline globules, most having ovoid nuclei, and several having spindle-shaped or giant nuclei all arranged fibrovacular stalks [68,72]. SPTs do not have mucus producing cells or extra-cellular mucus, differentiating them from MCN. They usually have rare to absent mitoses [73] and do not have cellular atypia, differentiating them from mucinous cystadenocarcinoma [65]. If SPTs have malignancy, it is typically low grade without lymph node metastases [73] with excellent survival [5].

Genetically, SPTs have not been shown typically to harbor p53 or KRAS mutations [65]. Studies have implicated the Beta-catenin gene (*CTNNB1*) and Wnt signaling in the pathogenesis of SPT, and several studies demonstrated that 100% of SPT harbored *CTNNB1* mutations [49,63]. In contrast, only 10% of lesions studied harbored a p53 mutation. This group found that with 100% sensitivity and specificity presence of *CTNNB1* mutation and the absence of *KRAS, GNAS*, or *RNF43* or chromosome 18 LOH. In regards to LOH, one study found that the number of point mutations was lower than any other pancreatic cyst types and less than any tumor evaluated by genome-wide sequencing to date [49]. Additionally, a majority of SPT also had aneuploidy, involving 16p and less frequently 11p [63].

## 5. Cystic Neuroendocrine Tumors (Cystic-NET)

Cystic neuroendocrine tumors are estimated to be less than 10% of PCLs [74,75,76,77,78]. They occur equally in men and women [74,78,79] in the 4th to 6th decades of life [78]. They are typically non-functional, present in the neck, body or tail of the pancreas, and their presence is a negative predictor of carcinoma, in contrast with non-cystic pancreatic neuroendocrine tumors [74,78,80]. One study that directly compared clinical characteristics of cystic versus solid neuroendocrine tumors found that cystic-NETs were statistically significantly more likely to be in men and solid tumors were more likely to demonstrate malignant behavior [77]. Overall 5-year survival following resection of cystic-NETs is excellent, with a recent systematic review calculating 92% overall 5-year survival. While a statistically significant difference in invasive carcinoma rates was found between solid-NET and cystic NET (34.1% versus 9.7%, *p* = 0.011), no difference was found in overall 5-year survival between solid-NET and cystic-NET. There was also no difference in recurrence rates found between solid-NET and cystic-NET (7.5–56.6% versus 4.8–28.6%, *p* = 0.24). The author of these findings noted this could be due to small sample size and limited, short-term follow up data obtained in their review [81].

Imaging is unable to reliably differentiate cystic-NETs from other PCLs as well as simple cysts, pseudocysts or SCAs [77,82]. Thus, EUS with fine needle aspiration and immunostaining has been suggested as a more effective modality for diagnosing cystic-NETs [77,83].

Cystic-NETs have been shown to stain positive for synaptophysin and chromogranin [75,77,79,83,84]. This staining helps distinguish Cystic-NETs from other PCLs that may have similar histological or immunohistochemical features. Cystic-NETs can be confused with cystic transformation of PDAC, however, cytology and staining patterns, lack of mucin, intra-luminal tumoral necrosis and nuclear pleomorphism make the distinction. Similarly, cystic-NETs differ from MCNs and IPMNs based on immunostaining and lack of mucin. In regards to differentiating between SPTs which typically do not stain with synaptophysin, SPTs can be further delineated from cystic-NETs by staining keratin negative, beta-catenin and trypsin positive consistent with acinar carcinomas, opposite from endocrine neoplasms. Since on imaging oligocystic serous cystadenomas can mimic cystic-NETs, they too can be differentiated by the presence of glycogen and clear cytoplasm which are absent in cystic-NETs [85].

Genetically, there is some debate regarding the pathophysiology of cystic-NETs. Associations with familial genetic syndromes such as multiple endocrine neoplasia type 1 (MEN1), von Hippel–Lindau syndrome (VHL), and neurofibromatosis-1 (NF1) have been described [86]. Bordeianou et al. showed cystic-NETs to be 3.5 times more likely in patients with MEN1 [75]. Furthermore, MEN1 associated cystic-NETs have been demonstrated to be multiple, in the tail and functioning [79]. However, the majority of studies have not found this association, suggesting cystic-NETs are sporadic, being larger than and resulting from cystic degeneration of solid, non-cystic pancreatic neuroendocrine tumors [75,82,83,84,87].

## 6. Conclusions

This review discussed the unique epidemiological, radiological, histological, molecular, and genetic aspects of PCLs with malignant potential including IPMN, MCN, SPT and cystic-NET. While malignant potential is highest in IPMNs, knowledge of the unique histologic, molecular and genetic characteristics of all four PCLs is helpful in risk stratification for these neoplastic lesions.

## Figures and Tables

**Table 1:** A summary of the different types of Intraductal Papillary Mucinous Neoplasms (IPMN; 15–33).

IPMN Type	Location in Pancreas	IPMN Duct Location	Mucin Expression	Invasion Rate	Invasive Subtype—%	Recurrence Rate	5-Year Survival
Gastric	Head (uncinate), periphery	BD & MD	MUC1-, MUC2-, MUC5AC+, MUC6+	9.4%	tubular—30.8%	7.9–15.3%	70–93.7%
Intestinal	Head	MD	MUC1-, MUC2+, MUC5AC+, CDX2+	41.6%	colloid—55.6%	19.8–24.4%	86.6–88.6%
Pancreatico-biliary	Head	MD	MUC1+, MUC2-, MUC5AC+, CDX2-	63.2%	tubular—90.2%	31.6–70.7%	35.6–52.0%
Oncocytic	Head	MD	MUC1+, MUC2-, MUC5AC+	45.8%	oncocytic—75%	12.5–25%	75–100%

BD: Branch Duct; MD: Main Duct.

## Supplementary Materials

The following are available online at http://www.mdpi.com/2079-9721/6/2/50/s1.
