# Supplementary material for: Pancreatic Cystic Lesions: Pathogenesis and Malignant Potential
Source: Diseases. 2018 Jun 13;6(2):50. doi: 10.3390/diseases6020050 (PMC6023528; doi:10.3390/diseases6020050)
Supplement: Supplementary file 1 [file diseases-06-00050-s001.pdf]

PCL type	% of PCLs	Epidemiology: Gender and Age	Location in pancreas	Histological features	Immunohistochemical features	Genomic mutations	Relative Invasion Rate	Recurrence following resection	Overall 5 year survival rate
IPMN	21-33%	Slightly more in men; 6th-7th decade	Head	Gastric, intestinal, pancreaticobiliary & oncocytic types (see Table 1 for further details)	Mucin (see Table 1 for further details)	KRAS Codon 12; GNAS Codon 201	27.6%-68%	0-41.7%	40-60% (see Table 1 for rates by type)
MCN	10%	Predominately in women; 4th decade	Body or tail	Ovarian-type storma	Mucin: 40% are MUC2+, CDX2+	KRAS codon 12; RNF43	17%	4.5%	75-93%
SPT	5%	Predominately in women; 3rd decade	Head or tail	Solid, pseudopapillary and/or hemorrhagic pseudocystic structures, PAS positive	Negative for mucin, synaptophysin, keratin; Positive for A1AT, NSE, vimentin, beta-catenin & trypsin	CTNNB1; aneuploidy, involving 16p, 11p	8-20%	10-15%	95-100%
Cystic-NET	10%	Equal in men & women; 4th-6th decade	Neck, body or tail	Cystic neoplasm; lack of intra-luminal tumoral necrosis & nuclear pleomorphism	Negative for mucin; Positive for synaptophysin & chromogranin	Sporadic,; association with MEN1, VHL, & NF-1	9.7%	4.8%-28.6%	92%

TABLE 2: A summary of the different types of PCLs (10-86)
